# Evidenzgenerierung und methodische Beratung durch das Projekt „EVAluationsforschung auf der Grundlage von Daten aus der klinischen Routineversorgung für die Medizininformatik-Initiative“ (EVA4MII)

**DOI:** 10.1007/s00103-026-04243-5

**Published:** 2026-05-04

**Authors:** Michelle Pfaffenlehner, Miriam Kesselmeier, Kai Günther, Kathrin Ungethüm, Viktoria Rücker, Flavia Remo, Harald Binder, André Scherag, Peter Heuschmann, Nadine Binder

**Affiliations:** 1 Institut für Medizinische Biometrie und Statistik, Universitätsklinikum Freiburg, Medizinische Fakultät, Universität Freiburg, Stefan-Meier-Straße 26, 79104 Freiburg im Breisgau, Deutschland; 2 Freiburger Zentrum für Datenanalyse, Modellbildung und AI, Universität Freiburg, Freiburg im Breisgau, Deutschland; 3 Institut für Medizinische Statistik, Informatik und Datenwissenschaften (IMSID), Universitätsklinikum Jena & Friedrich-Schiller-Universität Jena, Jena, Deutschland; 4 Institut für medizinische Datenwissenschaften (ImDS), Universitätsklinikum Würzburg, Würzburg, Deutschland; 5 Institut für Klinische Epidemiologie und Biometrie, Julius-Maximilians-Universität Würzburg, Würzburg, Deutschland; 6 Zentrale für Klinische Studien, Universitätsklinikum Jena, Jena, Deutschland; 7 Zentrale für Klinische Studien, Universitätsklinikum Würzburg, Würzburg, Deutschland; 8 Institut für Allgemeinmedizin, Universitätsklinikum Freiburg, Medizinische Fakultät, Universität Freiburg, Freiburg im Breisgau, Deutschland

**Keywords:** Elektronische Patientenakte, Routinedaten, Evaluationsforschung, Forschungsprozess, Beratung, Electronic health records, Routine data, Evaluation research, Research cycle, Consulting

## Abstract

Routinedaten aus der medizinischen Versorgung gewinnen für die Evidenzgenerierung an Bedeutung. In Deutschland wurden hierfür in den letzten Jahren neue Strukturen geschaffen. Beispielsweise wurden im Zuge der Medizininformatik-Initiative (MII) an allen Universitätskliniken Datenintegrationszentren (DIZ) aufgebaut, in denen Patient*innendaten pseudonymisiert, standardisiert und operationalisiert vorgehalten werden. Da Routinedaten, anders als Primärdaten, für Versorgungszwecke erhoben werden, müssen Aspekte der ursprünglichen Datenerhebung bei der Forschungsfrage, Studienplanung und -auswertung sowie bei der Interpretation der Ergebnisse berücksichtigt werden.

Das Projekt EVA4MII (EVAluationsforschung auf der Grundlage von Daten aus der klinischen Routineversorgung 4 MII) unterstützt Forschende bei der Analyse deutschlandweiter klinischer Routinedaten unter anderem durch Weiterbildungsangebote und eine zentrale Beratungsplattform. Die Beratung erfolgt durch ein interdisziplinäres Team mit methodisch-statistischer, datentechnischer und klinisch-epidemiologischer Expertise in enger Abstimmung mit datenbereitstellenden Einrichtungen. Das Angebot umfasst den gesamten Forschungsprozess – von der Studienplanung und den Formalitäten über Durchführung, Auswertung und Bewertung bis hin zur Veröffentlichung – und richtet sich an Projekte der MII und darüber hinaus.

Ziel des Artikels ist es, die Bedeutung methodischer Unterstützung bei der Analyse klinischer Routinedaten darzustellen und zentrale Punkte im Forschungsprozess zu identifizieren, an denen diese besonders relevant ist. Abschließend wird ein Ausblick auf zukünftigen Beratungsbedarf gegeben, wobei auch der Einsatz künstlicher Intelligenz als unterstützendes Werkzeug berücksichtigt wird.

## Abstract (en)

Routine data from healthcare are gaining importance for evidence generation. In Germany, new structures have been established in recent years to support their use. As part of the Medical Informatics Initiative (MII), data integration centres (DIZ) have been set up at all university hospitals, where patient data are made available in a pseudonymized, standardized and operationalized form. Since routine data, unlike primary data, are collected for healthcare purposes, aspects of the original data collection must be considered when formulating the research question, planning and analysing the study, and interpreting the results.

The EVAluation research based on data from routine clinical care 4 the MII (EVA4MII) project supports researchers in analysing nationwide clinical routine data, for example through training programs and a central advisory service. This support is provided by an interdisciplinary team with methodological-statistical, data-technical, and clinical-epidemiological expertise in close coordination with data-providing institutions. The service covers the entire research process, from study planning and formal requirements to implementation, analysis, evaluation, and publication, and is available to projects within the MII and beyond.

The aim of this article is to highlight the importance of methodological support in the analysis of clinical routine data and to identify key stages in the research process where such support is particularly relevant. Finally, an outlook on future advisory needs is provided, including the potential role of artificial intelligence as a supportive tool.

## Einleitung

Daten aus dem klinischen und ambulanten Versorgungsalltag von Patient*innen gewinnen für die Erforschung und Bewertung klinischer Fragestellungen zunehmend an Bedeutung. Dazu tragen insbesondere Großinitiativen wie die vom Bundesministerium für Forschung, Technologie und Raumfahrt (BMFTR) geförderte Medizininformatik-Initiative (MII; [1]) sowie strukturelle Weiterentwicklungen wie die Einführung der sektorenübergreifenden elektronischen Patientenakte (ePA) „ePA für alle“ in Deutschland [2] bei. Die Ergänzung von Evidenz aus klinischen Studien durch Versorgungsdaten ermöglicht eine realitätsnahe Beurteilung von Krankheitsverläufen und Interventionen. Erkenntnisse aus der Analyse dieser Real-World-Daten (RWD) werden als Real-World-Evidenz (RWE) bezeichnet.

Zu den Quellen von RWD zählen unter anderem Versorgungsdaten aus ePAs, die in Krankenhäusern, hausärztlichen Praxen oder anderen Gesundheitseinrichtungen dokumentiert werden. Weitere relevante Quellen sind Registerdaten, ambulante oder stationäre Abrechnungsdaten, Berichte von Patient*innen in Form von „patient reported outcome measures“ (PROMs), Biobanken sowie Daten, die durch (medizinische) Geräte wie Smartwatches, Blutdruckgeräte oder Patches, beispielsweise im Rahmen des Diabetesmanagements, erhoben werden [3–5].

Einen wichtigen Bestandteil der RWD stellen insbesondere Routinedaten aus der klinischen Versorgung dar, beispielsweise jene der deutschen Universitätskliniken. Um diese für Forschungszwecke leichter zugänglich und nutzbar zu machen, wurden im Zuge der MII an jedem Universitätsklinikum Deutschlands Datenintegrationszentren (DIZ; [6]) eingerichtet, in welchen die erhobenen Daten der Patient*innen aus dem jeweiligen Krankenhausinformationssystem (KIS) pseudonymisiert, standardisiert und operationalisiert im Format HL7® FHIR® (Health Level 7 *F*ast *H*ealthcare *I*nteroperable *R*esources [7]; nachfolgend als FHIR bezeichnet) gespeichert werden. Der Umfang und die Struktur der Daten werden im Kerndatensatz (KDS) der MII definiert und sind von jedem DIZ für alle Patient*innen bereitzuhalten [8].

Obwohl mit der MII eine standardisierte und forschungsfreundliche Infrastruktur geschaffen wurde, bleiben bei der Nutzung von Routinedaten für wissenschaftliche Analysen methodische und strukturelle Herausforderungen bestehen. Neben klassischen Herausforderungen von Beobachtungsstudien, wie Confounding und Bias, ergeben sich bei der Sekundärnutzung der Daten für Forschungszwecke zusätzliche Hürden. Diese betreffen in erster Linie die Qualität der Daten, welche sorgfältig für die jeweilige Forschungsfrage ermittelt werden muss. So ist die Operationalisierbarkeit bestimmter Variablen häufig durch eingeschränkte Datenverfügbarkeit (z. B. selektive Labordaten) oder kodierungsbedingte Limitierungen (z. B. bei Diagnosen durch Fragen der Abrechnungen oder Medikationen durch eine aktuell noch beschränkte digitalisierte Dokumentation) beeinträchtigt, was die Validität von Analysen einschränken kann (siehe z. B. [9]). Diese Herausforderungen, welche wir in einem Rapid-Review systematisch aufgearbeitet haben [10], verdeutlichen, dass die wissenschaftliche Nutzung von Routinedaten eine fundierte Auseinandersetzung mit deren technischen, klinischen und statistischen Eigenschaften erfordert. Dies setzt sowohl medizinische Fachexpertise als auch ein fundiertes Verständnis der Datengenerierung, -struktur sowie der daraus ableitbaren Implikationen für Studiendesign, Analyse und Berichterstattung der Ergebnisse voraus.

Die methodischen Leitlinien zum Umgang mit Routinedaten wurden zwar in den letzten Jahren kontinuierlich weiterentwickelt [11, 12] und die Verfügbarkeit versorgungsnaher Daten hat zugenommen. Dennoch bestehen im deutschen Evidenzökosystem weiterhin Lücken, insbesondere in Bezug auf die adäquate Anwendung vorhandener Methoden. Obwohl in vielen Fällen geeignete Methoden zur Verfügung stehen, fehlt es häufig an der notwendigen Unterstützung, um diese Methoden korrekt, kontextsensibel und ressourcenschonend umzusetzen. Um diese Lücke zu schließen, wurde im Rahmen des MII-Modul-2b-Projekts EVA4MII (EVAluationsforschung auf der Grundlage von Daten aus der klinischen Routineversorgung 4 MII) eine interdisziplinäre Beratungsplattform etabliert. Diese vereint methodisch-statistische, datentechnische und klinisch-epidemiologische Expertise und unterstützt Akteur*innen gezielt bei Planung, Durchführung und Bewertung von Studien auf Basis von Routinedaten.

Ziel dieses Artikels ist es, die Relevanz der methodischen Unterstützung bei der Analyse klinischer Routinedaten hervorzuheben und aufzuzeigen, an welchen Stellen des Forschungsprozesses eine solche Unterstützung besonders hilfreich sein kann. Zunächst werden die neuen Strukturen für die studienbasierte Routinedatenanalyse und das Projekt EVA4MII näher vorgestellt. Darauf aufbauend wird die Relevanz methodischer Beratung – mit speziellem Fokus auf MII-Daten – an den Schlüsselstellen des Forschungsprozesses verdeutlicht. Abschließend wird diskutiert, inwieweit künstliche Intelligenz (KI) zukünftig eine unterstützende Rolle in der methodischen Beratung einnehmen könnte.

## Neue Strukturen für studienbasierte Routinedatenanalysen

**Abb. 1:**
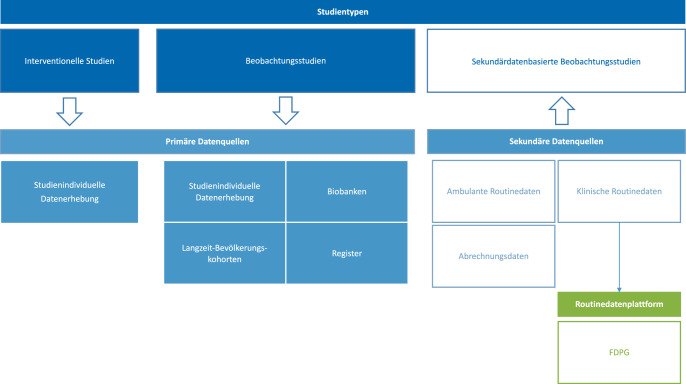
Sekundärdatenbasierte Beobachtungsstudien als neue Form der Evidenzgenerierung. Das Forschungsdatenportal für Gesundheit (FDPG) dient als zentrale Anlaufstelle für den Zugang zu Routinedaten und zur Übersicht von Datenbeständen

Randomisierte kontrollierte Studien (RCTs) gelten als Goldstandard zur Bewertung von Therapieeffekten, sind jedoch nicht immer durchführbar – etwa aus ethischen Gründen (z. B. bei vulnerablen Gruppen oder fehlender Vergleichstherapie) oder aufgrund zeitlicher, wirtschaftlicher oder organisatorischer Einschränkungen [13]. In solchen Fällen stellen Beobachtungsstudien wie prospektive oder retrospektive Kohortenstudien, Fall-Kontroll-Studien oder Querschnittsstudien eine Alternative dar [14]. Darüber hinaus können Beobachtungsstudien als Ausgangspunkt für die Hypothesengenerierung genutzt werden, indem sie potenzielle Zusammenhänge aufdecken, die anschließend im Rahmen (konfirmatorischer) Studien mit strengeren, randomisierten Designs überprüft werden. Bei interventionellen Studien werden die Daten i. d. R. studienindividuell erhoben und bisher nur selten mit bestehenden Datenquellen (z. B. Routine‑, Registerdaten) verknüpft. In Beobachtungsstudien hat die Nutzung von Primärdatenquellen eine größere Tradition als die Nutzung sekundärer Datenquellen, die ursprünglich nicht für Forschungszwecke erhoben werden. Sekundärdatenbasierte Beobachtungsstudien stellen eine vergleichsweise neuartige Form der Evidenzgenerierung dar, da sie auf bereits existierenden Daten basieren und sich damit von interventionellen sowie klassischen Beobachtungsstudien unterscheiden, bei denen Daten gezielt für eine Forschungsfrage erhoben werden (Abb. 1). Insbesondere zur Abbildung des realen Versorgungsalltags von Patient*innen sind Sekundärdaten von hoher Relevanz und erweitern die Erkenntnisse aus Studien mit Primärdatenerhebung [15].

Besonders nah an der Regelversorgung befinden sich dabei Krankenhausabrechnungsdaten, die jedes Krankenhaus in Deutschland an das Institut für das Entgeltsystem im Krankenhaus (InEK) übermitteln muss. Da diese Daten primär zur Abrechnung erhoben werden, enthalten sie häufig nur zusammengefasste Aufenthaltsinformationen (z. B. in Form von DRGs – „diagnosis-related groups“), was den Detailgrad der Informationen, etwa im Hinblick auf zeitliche Abläufe oder eine grobe Kodierung, und die Validität für bestimmte wissenschaftliche Fragestellungen einschränken kann [9, 16].

Darüber hinaus zählen insbesondere Daten der klinischen Versorgung, die aus Krankenhausinformationssystemen (KIS) stammen, zu sekundären Datenquellen. Diese Daten können zwar detaillierter als Abrechnungsdaten sein, liegen jedoch häufig nicht in standardisierter Form vor, sind eigentumsrechtlich den Kliniken zugeordnet und erfordern eine aufwendige Aufbereitung, bevor sie für Forschungszwecke nutzbar sind. Die Verwendung dieser Daten in (multizentrischen) Studien ist auch durch datenschutzrechtliche Vorgaben reguliert, speziell im Hinblick auf Verknüpfung, Weitergabe und Zugriffsrechte, was eine institutionsübergreifende Nutzung zusätzlich erschwert.

Um diesen Herausforderungen bei der Nutzung von Daten aus den KIS zu begegnen, wurden im Rahmen der MII die DIZ an allen Universitätskliniken in Deutschland eingerichtet. Als nationales Zugangsportal zum MII-KDS dient das *Forschungsdatenportal für Gesundheit* (FDPG; [17]), worüber Forschende die Routinedaten für ihre Forschungszwecke beantragen können. Dabei bietet das Portal einen strukturierten Überblick über die verfügbaren Datenbestände für die standortübergreifende Forschung und eine zentrale Koordination der Datenbereitstellung [17]. Nicht nur die Routinedaten der Universitätskliniken sollen über die MII für die Forschung nutzbar gemacht werden, sondern perspektivisch auch Daten aus Hausarztpraxen, Pflege- und Rehabilitationseinrichtungen sowie weiteren nichtuniversitären Krankenhäusern. Die durch das BMFTR geförderten *Digitalen FortschrittsHubs Gesundheit* knüpfen hierfür an die bestehenden MII-Strukturen an und erweitern diese gezielt in die regionale Versorgung. Sie schaffen damit eine zentrale Voraussetzung für die künftige Nutzung versorgungsnaher Daten in wissenschaftlichen Forschungsprojekten [18].

Durch die standardisierten Strukturen der MII mit der Errichtung des FDPG sind in Deutschland die Grundpfeiler für die Analyse medizinischer Routinedaten gelegt worden. Durch diese Initiative, im Rahmen derer die Daten aus diversen, technisch, strukturell und inhaltlich verschiedenen KIS und Systemen anderer Sektoren vereinheitlicht zur Verfügung gestellt werden, wurde insbesondere die Verwendung dieser Daten in multizentrischen bzw. deutschlandweiten Studien befördert. Zusammen mit dem *Gesundheitsdatennutzungsgesetz* (GDNG) soll damit die Versorgung verbessert und auch der Eingang dieser Daten in die Forschung erleichtert werden.

Wie sich mit diesen Daten nun sinnvoll und methodisch fundiert Forschung betreiben lässt, um die noch bestehende Lücke zwischen Datenverfügbarkeit und tatsächlicher Nutzung zu schließen, ist eine zentrale Fragestellung von EVA4MII. Dabei ist das Ziel, Forschungsprojekte individuell zu beraten und gleichzeitig methodisches Grundwissen zur Routinedatenanalyse verfügbar zu machen, um eine methodisch fundierte Nutzung von medizinischen Routinedaten zu erleichtern.

## Methodische Unterstützung bei der Routinedatenanalyse

**Abb. 2:**
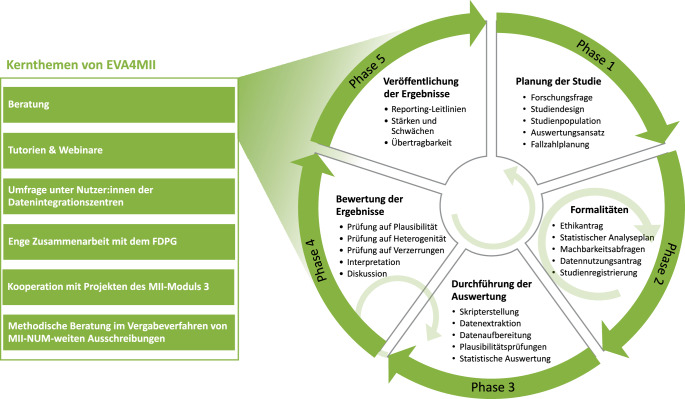
Kernthemen des Projektes EVA4MII (EVAluationsforschung auf der Grundlage von Daten aus der klinischen Routineversorgung für die Medizininformatik-Initiative (MII)) und die 5 Phasen des iterativen Forschungsprozesses, zu denen beraten werden kann. *FDPG* Forschungsdatenportal für Gesundheit, *NUM* Netzwerk Universitätsmedizin

Das MII-Modul-2b-Projekt EVA4MII macht es sich zur Aufgabe, Forschende bei der Analyse mit deutschlandweiten, klinischen Routinedaten zu unterstützen. Dieses Kooperationsprojekt der Universitätskliniken Würzburg (UKW), Jena (UKJ) und Freiburg (UKF) wird seit April 2023 vom BMFTR mit einer Laufzeit von 4 Jahren gefördert. Zusätzlich setzt sich EVA4MII zum Ziel, die Anforderungen an die in der Routine generierten und operationalisierten Daten u. a. für regulatorische Zwecke innerhalb Deutschlands zu ermitteln. Neben diesen Kernfunktionen beteiligt sich EVA4MII an einer Reihe weiterer Aktivitäten, die den nachhaltigen und qualitätssicheren Umgang mit (MII‑)​Routinedaten fördern sollen (Abb. 2, links). Dazu zählen die Entwicklung von Tutorien und Webinaren zur Orientierung, Weiterbildung und Information von Nutzenden der MII-Daten, die Erörterungen bisheriger Erfahrungen und Hindernisse bei der Analyse der MII-Daten mithilfe von Umfragen und die enge Zusammenarbeit mit dem FDPG. Diese Weiterbildungsressourcen können über die MII-Academy^1^ und die EVA4MII-Webseite^2^ kostenlos genutzt und beispielsweise in der Lehre der Studierenden eingesetzt werden.

Zudem stand EVA4MII bei den Ausschreibungen im Rahmen des MII-NUM (Netzwerk Universitätsmedizin) [19] in den Jahren 2024 und 2025 sowohl bei der Antragseinreichung als auch bei dem Vergabeverfahren beratend zur Seite. Bei diesen Ausschreibungen handelte es sich um die Förderung von Datennutzungsprojekten über das FDPG, um letztendlich eine Verbesserung der standardisierten Prozesse zu ermöglichen. Darüber hinaus kooperiert EVA4MII eng mit den Modul-3-Projekten der MII und berät diese.

Seit 2023 bietet EVA4MII eine zentrale Anlaufstelle für Beratungsanfragen und unterstützt Forschende entlang des gesamten Forschungsprozesses mit interdisziplinärer, methodischer Expertise bei der Analyse von Routinedaten. Das kostenlose Angebot richtet sich an Forschende, die MII-Daten nutzen, und versteht sich als unterstützende Beratung zur eigenständigen Umsetzung. Je nach Bedarf können ein oder mehrere Beratungstermine über ein Formular beantragt werden, die per E‑Mail oder Videokonferenz stattfinden. Weitere, aktuelle Informationen rund um das Beratungsangebot und wie davon Gebrauch gemacht werden kann, sind über die EVA4MII-Webseite verfügbar. Der Fokus der Beratung kann sich von der Definition einer Fragestellung über die Operationalisierung der Informationen und Endpunkte bis hin zur Planung einer standortübergreifenden Analyse der Daten erstrecken oder Teilaspekte davon abdecken (Abb. 2).

Die Beratungsfunktion setzt bereits in der frühen Planungsphase einer Studie an (Abb. 2, Phase 1). Die Entwicklung einer präzisen und fundierten Fragestellung ist schon vor der Beantragung der Daten von zentraler Bedeutung und wird daher auch als Grundprinzip in den „Empfehlungen zur guten epidemiologischen Praxis“ hervorgehoben [20]. Ein solches Vorgehen minimiert das Risiko einer fehlgeleiteten explorativen Datenauswertung, die zum Ziel hat, lediglich statistisch signifikante Ergebnisse zu produzieren (sog. Datenfischen bzw. HARKing [21, 22]). Für therapeutische Fragestellungen bieten sich das *PICO-Schema* bzw. *PECO-Schema* an, bei ätiologischen Forschungsfragen das *PEO-Schema*, wobei P für Patient*in, I für Intervention, E für Exposition (Exposure), C für Vergleich (Comparison) und O für Endpunkt (Outcome) stehen [23]. Die Wahl eines geeigneten Studiendesigns orientiert sich folglich an der Art der Fragestellung und Verfügbarkeit der Daten, insbesondere im Hinblick auf den zeitlichen Verlauf und die Struktur der Daten.

Eng mit der Fragestellung verbunden ist die Festlegung der Studienpopulation, die durch die Ein- und Ausschlusskriterien definiert wird. Um die Anzahl der verfügbaren Patient*innen bzw. die Fallzahl in der definierten Studienpopulation abzuschätzen, kann über das FDPG eine Machbarkeitsabfrage durchgeführt werden. Während dieses Vorgangs ist es möglich, für die ausgewählte Kohorte die Verfügbarkeit der Merkmale, die für die Beantwortung der Forschungsfrage erforderlich sind, zu überprüfen. Die Auswahl der notwendigen Variablen stellt eine weitere zentrale Herausforderung dar. Zum einen ist es ggf. nicht möglich, alle gewünschten Variablen über Routinedatenplattformen zu extrahieren. Zum anderen können ggf. nicht alle relevanten Variablen in der erforderlichen Definition aus den Daten abgeleitet oder erfasst werden. Besonders bei zeitlichen Abfolgen oder der Extraktion spezifischer Parameter kann es zwischen den Datenhaltenden (DIZ/Unikliniken) erhebliche Unterschiede geben. Laborwerte oder Angaben zu z. B. Gewicht und Größe sind häufig unvollständig oder nur für bestimmte Patientenpopulationen verfügbar. Ursachen können etwa klinische Abläufe oder fehlende Dokumentationen sein. Falls für die Auswertung zentrale Variablen nicht in geeigneter Weise im MII-KDS verfügbar sind, sollte eine Anpassung der ursprünglich geplanten Fragestellung in Betracht gezogen werden. Bei den hier nur kurz angerissenen Aspekten kann EVA4MII beratend zur Seite stehen. Nach der Projektplanung umfasst das Beratungsangebot auch die Unterstützung bei der Erstellung erforderlicher Dokumente im Rahmen der Routinedatennutzung (Abb. 2, Phase 2). Dazu gehört unter anderem die Erarbeitung des Ethikantrags und des damit verbundenen Studienprotokolls (inklusive Fallzahlabschätzung bzw. Poweranalyse) zur Beschreibung des Forschungsvorhabens, der Datennutzungsantrag für den Datenerhalt über das FDPG sowie der statistische Analyseplan zur detaillierten Festlegung der statistischen Auswertung. Hierbei kann der Beratungsfokus speziell auf der Formulierung der im Studienprotokoll festzuhaltenden Aspekte wie Hypothesen, Studienpopulation, Zielgrößen und geplante statistische Auswertungsmethoden gelegt werden. Dies umfasst neben dem statistischen Ansatz auch die Unterstützung bei der Entscheidung zwischen verteilter oder zentraler Analyse und deren Umsetzung sowie die Identifikation potenzieller Hindernisse bei der Variablendefinition und möglicher Quellen von Heterogenität. Dabei ist besonders zu beachten, dass Daten bei verteilten Analysen nicht dem Forschenden übergeben werden, sondern am jeweiligen Standort verbleiben. Dafür werden Analyseskripte erstellt, die zu den jeweiligen beteiligten Standorten gesendet und dort ausgeführt werden. Der Vorteil dieses Ansatzes ist, dass dabei alle Patient*innen, auch jene ohne sogenannten Broad Consent – also ohne die breite Patienteneinwilligung zur standortübergreifenden Nachnutzung der Daten aller universitätsmedizinischen Standorte der MII [1, 24] – in die Analyse eingeschlossen werden können. Im Rahmen einer verteilten Analyse ist die Bereitstellung von synthetischen Datensätzen, d. h. simulierten Daten, für die Skripterstellung und -erprobung hilfreich, da Forschende i. d. R. keinen direkten Zugriff auf die Rohdaten erhalten [25, 26].

Nach Abschluss der formalen Vorbereitungen beginnt Phase 3 des Forschungsprozesses, die Auswertung (Abb. 2). Bei Routinedatenanalysen mit zentraler Auswertung startet die Auswertung mit dem Erhalt der angeforderten Datensätze. Da die Daten im Rahmen der MII standardisiert im FHIR-Format bereitgestellt werden, bei dem es sich nicht um ein klassisches Tabellenformat handelt, müssen die Daten für die statistische Analyse i. d. R. zunächst in tabellarische Strukturen überführt werden – z. B. in R mittels *fhircrackr* [27] oder in Python mittels *FHIR-PYrate* [28]. Obwohl grundsätzlich auch eine direkte Analyse im FHIR-Format möglich ist, erfolgt in der Praxis meist eine tabellarische Aufbereitung, da gängige statistische Werkzeuge mit Tabellenstrukturen arbeiten. Erst im Anschluss können die entsprechend den Vorgaben im statistischen Analyseplan und auf Grundlage der weiteren in Phase 2 definierten Kriterien weiter aufbereitet und analysiert werden. Im Rahmen der Aufbereitung ist zu beachten, dass standortspezifische Unterschiede der KIS zu Unterschieden in der Befüllung der KDS-Ressourcen führen können. Zwar definiert die MII eine allgemeine Struktur der Datenbank, erlaubt aber Spielraum hinsichtlich des Formats und der Detailtiefe einzelner Items, damit die Struktur mit allen KIS kompatibel ist. Die standortspezifischen Besonderheiten umfassen u. a. die Kodierung von Medikamenten (z. B. Einnahme vs. Verordnung, ambulant vs. stationär), Diagnosen (in Deutschland häufig erst nach der Entlassung für Abrechnungszwecke erfasst) sowie Laborwerte (insbesondere im Hinblick auf Einheiten, Messmethoden und Verfügbarkeit). Im Rahmen der Datenaufbereitung ist daher eine Plausibilitätsprüfung empfehlenswert – beispielsweise bezüglich der Wertebereiche oder hinsichtlich eines Widerspruchs zwischen den Werten (auch bei Mehrfachmessungen) einer Person. In diesen Bereichen sowie insbesondere bei der statistischen Auswertung gibt es im EVA4MII-Team Expertise und Erfahrungen aus vergangenen und laufenden Projekten.

In Phase 4 des Forschungsprozesses steht die Bewertung der Ergebnisse im Fokus. Wird eine verteilte Analyse durchgeführt, erhalten Forschende die aggregierten Ergebnisse der beteiligten Standorte und müssen diese im Hinblick auf Plausibilität und Heterogenität prüfen. EVA4MII kann in diesem Schritt durch bereits gesammelte Erfahrungen Hilfestellung leisten, insbesondere durch die Einschätzung verteilter Analyseergebnisse. Unabhängig vom gewählten Analyseansatz – verteilt oder zentral – bergen Beobachtungsstudien im Allgemeinen ein Risiko für systematische Verzerrungen und Confounding. Diese Einflüsse sollten bereits durch die Wahl eines geeigneten Studiendesigns und der Methoden minimiert und zusätzlich durch Sensitivitätsanalysen überprüft werden. Dennoch ist es insbesondere bei verteilten Analysen ratsam, sowohl die lokalen Ergebnisse als auch die Ergebnisse aus der dazugehörigen Metaanalyse auf Plausibilität zu prüfen, um mögliche weitere Verzerrungsquellen sowie Aspekte der Datenqualität und -vollständigkeit zu adressieren und gegebenenfalls zu diskutieren. Die Prüfung kann durch Literaturabgleich bzw. durch den Vergleich mit den eigenen Erfahrungen und Erwartungen erfolgen. Mögliche Ursachen müssen im Nachgang ergründet werden. Denkbare Ursachen für abweichende Ergebnisse können z. B. die verwendeten Primärsysteme an den Krankenhäusern, der lückenhafte Anschluss von Stationen oder – ganz klassisch – die Nichtberücksichtigung eines bekannten Confounders in der Auswertung sein. Wenn eine Berücksichtigung aufgrund zu vieler fehlender Werte oder kompletten Fehlens in den Daten nicht möglich ist, dann muss das als Limitation klar aufgeführt werden. Durch diese Plausibilitätsprüfungen kann es geschehen, dass die Forschenden zu dem Schluss kommen, dass einige Phasen des Forschungsprozesses wiederholt werden müssen, um auf die gewonnenen Erkenntnisse entsprechend reagieren zu können. Das kann insbesondere auch die ursprüngliche Planung der Studie beeinflussen, da Änderungen über mehrere Phasen notwendig werden könnten.

Letztlich sind das Zusammentragen der Analyseergebnisse und deren Interpretation, insbesondere im Hinblick auf Verzerrungsquellen und Limitationen, entscheidend für die Publikation der Studie, was im Forschungsprozess den letzten wichtigen Schritt darstellt (Abb. 2, Phase 5). Auch bei diesem Aspekt kann eine Beratung durch EVA4MII helfen. Für das Berichten von Studienergebnissen gibt es darüber hinaus bereits diverse Leitlinien [11, 29].

## Zukünftiger Beratungsbedarf

Die Nutzung von Routinedaten für Evidenzgenerierung steht in Deutschland noch am Anfang, zeigt aber bereits eine große Vielfalt an Anwendungsmöglichkeiten. Die Erweiterung der klinischen MII-Daten durch die Regelversorgungsdaten aus Hausarztpraxen oder anderen Gesundheitseinrichtungen und damit einhergehende neue Herausforderungen für Forschende werden dabei den skizzierten Beratungsbedarf noch vergrößern. Durch die stetige Weiterentwicklung und Verbesserung der MII-Daten wird eine Plattform wie EVA4MII zukünftig auch bei der Planung von komplexen Interventionsstudien beraten können, beispielsweise wenn Routinedaten als Kontrollen dienen, primäre und sekundäre Daten verknüpft werden oder Sekundärdaten zur Erhebung und Verlängerung des Follow-ups eingesetzt werden [30].

Darüber hinaus kann eine Beratungsplattform wie EVA4MII Forschende auch bei der Einwerbung von Drittmitteln sowie beim Verfassen von Studienanträgen für komplexe Interventionen gezielt beraten und unterstützen. Vor diesem Hintergrund halten wir auch künftig das Beratungsangebot von EVA4MII für Forschende, die Routinedatenanalysen mit Daten aus den DIZ durchführen möchten, für sinnvoll. EVA4MII dient als eine zentrale Anlaufstelle für methodische Beratungen bezüglich der Nutzbarkeit von Routinedaten der DIZ zu definierten Forschungszwecken. Das Beratungsangebot bündelt die für Routinedatenanalysen erforderlichen methodischen Expertisen, die kontinuierlich um spezifisches Wissen zu den Besonderheiten dieser Daten erweitert werden und damit allen Forschenden zugutekommt.

Dieses Beratungsangebot ist komplementär zu anderen Beratungsangeboten in der Universitätsmedizin, wie z. B. das der Koordinierungszentren für Klinische Studien (KKS)/Zentren für Klinische Studien (ZKS) an den einzelnen Standorten, die schwerpunktmäßig bei regulierten Studien beraten, sowie zu anderen methodischen Beratungseinheiten. Zu Letzteren zählen z. B. methodische Institute der Universitätsmedizin oder Angebote im Rahmen des Netzwerks Universitätsmedizin (z. B. NUM Methoden- und Bioprobenhub), die auf prospektiv geplante klinisch-epidemiologische Studien oder Linkage mit versorgungsnahen Daten ausgerichtet sind. Die Weiterbildungsressourcen von EVA4MII können auch lokalen Beratungsinstituten der Universitätsmedizin zugutekommen und Forschende in Zusammenarbeit mit EVA4MII beratend begleiten. Unabhängig von der Art der genutzten Datenquellen ist es generell sinnvoll und empfehlenswert, eine statistisch-methodische Beratung bereits in der Planungsphase des Forschungsprojekts in Anspruch zu nehmen.

Die Förderung von EVA4MII im Rahmen der MII ist bis März 2027 zeitlich befristet. Aus diesem Grund sollten bereits jetzt Konzepte für eine nachhaltige Verankerung der etablierten Beratungsangebote diskutiert werden.

### Künstliche Intelligenz und methodische Beratung

Mit dem rasanten Fortschritt im Bereich der KI gewinnen KI-gestützte statistische Auswertungsverfahren zunehmend an Bedeutung. Vor allem Angebote auf Basis von generativen Sprachmodellen, wie beispielsweise ChatGPT von OpenAI, gewinnen derzeit an Popularität, werden immer leistungsfähiger und sind einfach zu bedienen. Die generierten Antworten und vorgeschlagenen Methoden benötigen jedoch eine Bewertung hinsichtlich ihrer Sinnhaftigkeit in Bezug auf das Forschungsvorhaben und die zugrunde liegenden Daten. Forschende ohne einschlägige Kenntnisse in Statistik und Programmierung sowie ohne Wissen um die Datenerhebung und entsprechende klinische Hintergründe sind potenziell nur eingeschränkt in der Lage, die generierten Antworten kritisch und angemessen zu hinterfragen und sie dann auch adäquat umzusetzen. Daher ist es notwendig, KI-generierte Informationen mithilfe methodischer und statistischer Expertise, beispielsweise über eine Beratung durch EVA4MII, zu bewerten und aufzuarbeiten. In diesem Zusammenhang sollte die EXPOLS-Studie [31] erwähnt werden, in der das Potenzial der Integration von Large-Language-Modellen (LLMs) in die statistische Beratung untersucht wird. Dabei soll sowohl die Perspektive der Beratenden als auch der Ratsuchenden berücksichtigt werden. In einer weiteren Studie wurde ferner die Nützlichkeit von LLMs für typische Aufgaben von Biostatistiker*innen anhand konkreter Anwendungsfälle untersucht, um zu zeigen, wie generative KI sinnvoll in den täglichen Arbeitsablauf integriert werden kann [32]. Die Autor*innen warnen vor einer unkritischen Nutzung, betonen jedoch das Potenzial bei reflektierter Anwendung, da LLMs auf Fehlerhinweise oft selbstständig reagieren. Zudem präsentieren sie eine Leitlinie für deren Einsatz.

Neben fachlich-inhaltlichen Aspekten ist bei der Auswertung sensibler Gesundheitsdaten die Einhaltung des Datenschutzes essenziell. Vor diesem Hintergrund stellt sich auch die Frage, inwieweit der Einsatz von Chatbots hier zulässig ist [33]. In der Regel werden diese dialogisch benutzt, doch es besteht bereits die Möglichkeit, per einfacher Prompteingabe Analysen zu medizinischen Fragestellungen auf hochgeladenen Datensätzen durchzuführen. Neben Themen des Datenschutzes werden auch hier Fragen in Bezug auf die Vertrauenswürdigkeit der Ergebnisse berührt. Eine Lösung für Datenschutzbedenken könnten lokal implementierte LLMs darstellen.

In jedem Fall sollten die Verwendung von KI und der Rückgriff auf statistische Beratung nicht als Widerspruch begriffen werden. KI kann bei entsprechender Anwendung ein probates Hilfsmittel und eine Erleichterung darstellen, ersetzt jedoch die menschliche Intelligenz bei der Durchführung von Studien derzeit nicht [32].

## Fazit

Mit den neu etablierten deutschlandweiten Strukturen, insbesondere im Rahmen der MII, wurden die Voraussetzungen für die Nutzung elektronisch erfasster klinischer Routinedaten zur Generierung von Evidenz geschaffen. Die effektive Nutzung dieser Daten bleibt eine methodische und organisatorische Herausforderung. Das Projekt EVA4MII adressiert diese Lücke gezielt über eine Beratungsplattform sowie die Entwicklung gezielter Weiterbildungsmaßnahmen. Im Gegensatz zu Studien mit primärer Datenerhebung verläuft der Forschungsprozess bei der Nutzung von Routinedaten dynamisch. Aufgrund der spezifischen Eigenschaften dieser Daten kann es notwendig sein, in verschiedenen Phasen des Prozesses einen Schritt zurückzugehen und den Prozess erneut zu durchlaufen. Die Analyse von Routinedaten erfordert dabei nicht nur methodische Unterstützung entlang des gesamten Forschungsprozesses, sondern auch ein interdisziplinäres Team, das Verständnis für Programmierung, Statistik, Medizin und die Daten aufweist, wobei hier bis zu einem gewissen Grad KI als weiteres Hilfsmittel einbezogen werden kann.

## Footnotes

1 https://www.medizininformatik-initiative.de/de/konsortien/mii-academy.

2 www.ukw.de/eva4mii.
